# Post-Diagnosis Adherence to the Mediterranean Diet and Cancer Recurrence and Fatigue Outcomes in Cancer Survivors, with Emphasis on Colorectal Cancer: A Systematic Review and Meta-Analysis

**DOI:** 10.3390/nu18050807

**Published:** 2026-02-28

**Authors:** Dimitris Papamichael, Kyriacos Felekkis, Eleni P. Andreou

**Affiliations:** Department of Life Sciences, School of Life and Health Sciences, University of Nicosia, 46 Makedonitisas Avenue, Nicosia CY1700, Cyprus; ditris_pap@hotmail.com (D.P.); felekkis.k@unic.ac.cy (K.F.)

**Keywords:** colorectal cancer, mediterranean diet, cancer recurrence, cancer survivors, cancer-related fatigue, nutrition, survivorship

## Abstract

Background: Cancer survivors face heightened risks of recurrence and persistent cancer-related fatigue (CRF), both of which impair quality of life. The Mediterranean Diet (MD), characterized by its antioxidant and anti-inflammatory profile, has been proposed as a potentially beneficial dietary pattern. This systematic review and meta-analysis evaluated the association between post-diagnosis MD adherence and outcomes of cancer recurrence and CRF among adult survivors, with particular attention to colorectal cancer. Methods: Systematic searches of PubMed, CINAHL, and the Cochrane Library (January 1995–May 2024) identified prospective cohort studies and randomized controlled trials (RCTs) reporting post-diagnosis MD adherence. Primary outcomes were cancer recurrence and CRF. Random-effects models were applied for recurrence analyses due to anticipated heterogeneity, while a fixed-effects model was used for CRF given the limited number of trials. Heterogeneity was assessed using the *I*^2^ statistic. Risk of bias was evaluated using the Cochrane RoB 2 tool (RCTs) and ROBINS-I (cohort studies). This review was registered in PROSPERO (CRD420251248086). Results: Eight studies met inclusion criteria: six prospective cohort studies assessed recurrence (*n* = 6697), and two RCTs assessed CRF (*n* = 76). For recurrence, higher post-diagnosis MD adherence was associated with a lower hazard of recurrence or cancer-specific mortality (HR = 0.83; 95% CI: 0.70–0.99; *I*^2^ = 49%). For CRF, the pooled effect from two independent RCTs showed no statistically significant overall effect (MD = 0.29; 95% CI: −0.58 to 1.16). Both outcomes were limited by small study numbers and methodological heterogeneity. Conclusions: Higher adherence to the Mediterranean Diet after cancer diagnosis was associated with recurrence-related outcomes in observational studies, while evidence for CRF remains exploratory and statistically imprecise. Larger, adequately powered randomized trials are needed to clarify the role of the Mediterranean Diet in survivorship care.

## 1. Introduction

Five-year survival rates for colorectal cancer have improved substantially over time, particularly in countries with established screening and treatment programs, although survival remains strongly dependent on stage at diagnosis [1,2]. However, CRC survivors are at increased risk of reduced quality of life, sleep disturbances [3], fatigue [3,4], and cancer recurrence (CR) [5].

Cancer recurrence is believed to be due to micrometastatic clones of the original tumor that was removed, with the recurrence potentially being local or manifesting in other organs and locations [6]. The 5-year recurrence risk following curative-intent surgery for colorectal cancer ranges from approximately 30% in patients with stage II–III disease to substantially higher rates in advanced-stage cases treated with surgery alone. Most recurrences occur within the first 1–2 years after surgery. These percentages decrease to 6–30% when adjuvant chemotherapy is also administered [7]. According to data from the Global Cancer Observatory (GLOBOCAN), the average time to recurrence is approximately 1.1 years post-surgery [8].

In addition to chemotherapy, several studies suggest that the risk of colorectal cancer recurrence can be reduced through the use of nonsteroidal anti-inflammatory drugs (NSAIDs) [9] and/or omega-3 polyunsaturated fatty acids [10]. However, long-term use of NSAIDs increases cardiovascular risk, and the efficacy of omega-3 supplementation has not been confirmed. For this reason, it is essential to identify alternative strategies with greater efficacy and fewer side effects [11].

On the other hand, cancer-related fatigue (CRF) is a common complication among patients, with epidemiological data showing that 41% of individuals who have had colorectal cancer report experiencing it. Moreover, this symptom may persist for more than 5 years in this population and affect up to 35% of survivors [12,13]. People experiencing fatigue often face cognitive difficulties, early and persistent tiredness, hot flashes, reduced physical function, insomnia, depression, and significantly impaired quality of life [14,15]. Those who have undergone chemotherapy are particularly at risk [16,17]. Additional contributing factors include gender (women more than men), living alone, lower educational levels, and the presence of at least one comorbidity [18]. The pathophysiological mechanisms behind fatigue are not yet fully understood. Potential contributing factors include dysfunction of the hypothalamic–pituitary–adrenal (HPA) axis, circadian rhythm disruption, pro-inflammatory cytokines, skeletal muscle loss, and certain genotypes of the SLC6A4 gene [18,19].

Several biological mechanisms have been proposed to explain a potential role of the Mediterranean Diet (MD) in cancer survivorship. The MD is rich in polyphenols, omega-3 fatty acids, and fiber, which are associated with anti-inflammatory and antioxidant effects and may influence immune and metabolic pathways involved in cancer progression. For cancer-related fatigue, dysregulation of the hypothalamic–pituitary–adrenal (HPA) axis, chronic inflammation, and altered neurotransmitter activity have been implicated. However, these mechanisms remain largely hypothesized in colorectal cancer survivors, as direct interventional evidence linking Mediterranean Diet adherence to modulation of HPA-axis activity or neurotransmitter pathways in this population is currently limited. These mechanisms provide a biologically plausible basis for exploring MD adherence in cancer survivorship [15].

Treatment strategies for managing cancer-related fatigue (CRF) include a combination of pharmacological and non-pharmacological approaches. Pharmacological treatments are typically reserved for select cases and are generally recommended for short-term use, as their long-term efficacy remains unconfirmed. In contrast, psychosocial interventions play a central role and are most effective when they incorporate disease education, identification of potential underlying causes, psychoeducational support, and cognitive behavioral therapy. Additionally, physical activity is widely recognized as a beneficial strategy in reducing fatigue and improving quality of life among cancer survivors [20,21].

Lifestyle improvement—which includes maintaining a healthy weight, avoiding smoking, engaging in regular physical activity, adhering to a high-quality healthy diet, and moderating alcohol consumption—has been shown to reduce mortality risk among cancer survivors [22,23]. Additionally, adopting healthier eating habits after diagnosis decreases mortality risk, both overall and cancer-specific, regardless of dietary habits prior to diagnosis [24,25]. Thus, it is evident that dietary improvements after diagnosis are essential.

The American Institute for Cancer Research (AICR) provides the same nutritional guidelines for colorectal cancer survivors as for survivors of other types of cancer. However, it is acknowledged that the specific needs of this population may vary and warrant further study [26]. Despite the large number of colorectal cancer survivors, there are still no evidence-based nutritional guidelines specifically aimed at reducing fatigue or recurrence risk [27].

As summarized in a review by Inglis and colleagues, the diet of cancer survivors should include foods from all food groups. Emphasis should be placed on adequate protein intake to maintain or increase muscle mass, sufficient intake of polyunsaturated fatty acids, and increased consumption of complex carbohydrates rich in dietary fiber [15]. Moreover, guidelines from the AICR and the American Cancer Society (ACS) suggest that maintaining a healthy body weight and consuming a diet rich in fruits, vegetables, and whole grains—such as the Mediterranean Diet (MD)—may increase disease-free survival [28].

The MD is characterized as a dietary pattern rich in bioactive nutrients derived from food that can promote health both directly and through epigenetic mechanisms [29]. Its protective role against degenerative diseases is well-documented, with statistically significant findings across numerous studies. Several systematic reviews and meta-analyses have shown that it reduces the risk of cancer incidence and mortality in both healthy individuals and cancer patients [30,31,32].

Individuals adhering to the MD, especially those consuming polyphenol-rich foods, show lower levels of inflammation, which may be associated with reduced recurrence risk [33]. Greater adherence prior to diagnosis has also been associated with lower tumor aggressiveness [34] and better quality-adjusted life expectancy [35]. Furthermore, adopting even one dietary goal from the MD has been shown to reduce all-cause mortality by 12% over a 7-year period among colorectal cancer survivors [36]. Notably, the MD’s effectiveness in reducing cancer risk is not attributed to individual food components but rather to overall adherence to the dietary pattern [37].

Despite the recognized protective effects of the MD in cancer prevention and treatment outcomes, its specific impact on cancer recurrence risk and fatigue levels among colorectal cancer survivors has not been sufficiently studied. Additionally, heterogeneity in research protocols and methodologies contributes to conflicting conclusions regarding its effectiveness. Therefore, the aim of this systematic review and meta-analysis was to evaluate the impact of post-diagnosis adherence to the Mediterranean Diet on cancer recurrence and cancer-related fatigue among adult cancer survivors, with particular emphasis on colorectal cancer survivors, while incorporating evidence from other cancer types where colorectal cancer-specific data were limited.

### 1.1. Rationale

Colorectal cancer (CRC) is one of the most commonly diagnosed cancers worldwide. Advances in early detection and treatment have significantly improved survival outcomes, with over 80% of patients now surviving their initial diagnosis. However, survivorship often comes with new challenges. Among these, cancer recurrence remains a major concern, with rates ranging from 30% to 80%, depending on the cancer stage and treatment type—such as surgery alone versus combined with adjuvant chemotherapy. Additionally, cancer-related fatigue (CRF) is a prevalent and persistent symptom affecting quality of life, with physical, emotional, and cognitive consequences that may persist for years after treatment completion.

Emerging evidence emphasizes the role of lifestyle interventions in improving long-term outcomes for cancer survivors. In particular, the Mediterranean Diet (MD)—characterized by high intake of fruits, vegetables, whole grains, legumes, and healthy fats (especially olive oil) and moderate consumption of fish and dairy—has demonstrated protective effects against a variety of chronic diseases, including cardiovascular disease and several forms of cancer. Its anti-inflammatory and antioxidant properties suggest a plausible role in modulating biological pathways involved in cancer progression and recurrence.

While the MD’s role in cancer prevention is well-established, its impact on recurrence and CRF in CRC survivors remains underexplored. Given the limited number of CRC-specific interventional studies, this review also included evidence from other cancer types to provide a broader understanding. However, due to the heterogeneity in cancer types, results must be interpreted with caution. There is an urgent need for CRC-specific, high-quality trials evaluating the MD as a survivorship strategy.

### 1.2. Primary Aim and Justification

Given the lack of CRC-specific interventional studies, this review integrates broader evidence from other cancer survivor populations where necessary to inform CRC survivorship care. The aim of this systematic review and meta-analysis is to evaluate the impact of adherence to the Mediterranean Diet on cancer recurrence and fatigue levels among individuals who have survived cancer, with a specific focus on colorectal cancer survivors.

### 1.3. Objectives

(a) To assess the association between adherence to the Mediterranean Diet and the risk of cancer recurrence in cancer survivors, using data from prospective cohort and randomized controlled trials.
(b) To evaluate the effect of the Mediterranean Diet on levels of cancer-related fatigue (CRF) in cancer survivors.
(c) To identify variations in outcomes based on dietary adherence scores, patient characteristics, or study design.
(d) To explore and propose potential modifications to the Mediterranean Diet tailored specifically for colorectal cancer survivors based on observed evidence.

### 1.4. Hypothesis

1. Primary Hypothesis: Higher adherence to the Mediterranean Diet suggests a potential association with reduced risk of cancer recurrence in colorectal cancer survivors.
2. Secondary Hypothesis: Greater adherence to the Mediterranean Diet is associated with lower levels of cancer-related fatigue among colorectal cancer survivors.

## 2. Methodology

### 2.1. Protocol

This systematic review and meta-analysis was conducted in accordance with the PRISMA 2020 guidelines (Preferred Reporting Items for Systematic Reviews and Meta-Analyses) [38]. The Prisma 2020 checklist is submitted as Supplementary Materials File S1. A review protocol was developed prior to the commencement of the review, and it was registered in PROSPERO (CRD420251248086). This ensured that the methodology was vetted for scientific rigor and alignment with established systematic review standards.

### 2.2. Eligibility Criteria

This review included prospective cohort studies and randomized clinical trials involving adult cancer survivors. However, the definition of a cancer survivor is not yet consistent in the literature. Some studies define survivors as individuals immediately following diagnosis, others during treatment, and others after completion of treatment. For the purposes of this review, cancer survivors were defined as individuals who have completed their primary cancer treatment.

Nevertheless, studies in which participants had been diagnosed at least six months prior to enrollment—regardless of whether completion of treatment is explicitly mentioned—were also included. This criterion was applied because dietary habits often change after diagnosis, during treatment, and throughout recovery [25].

To be included, studies had to evaluate participants’ dietary habits after diagnosis. This ensured that dietary changes post-diagnosis, rather than pre-diagnosis habits, were captured. The review included studies assessing the impact of the Mediterranean Diet (MD), regardless of the specific dietary protocol used, in the eligible population. Additionally, studies examining individual foods or nutrients that align with the principles of the MD were also included.

Intervention studies had to include a control group, which could consist of either other cancer survivors or healthy individuals. If cancer survivors served as the control group, they had to receive standard care or another dietary intervention different from the MD. The outcomes evaluated included the risk of cancer recurrence and the level of cancer-related fatigue. Studies that did not assess either of these outcomes were excluded.

It is important to note that the included studies varied in how they defined a “cancer survivor.” Some considered survivorship from the point of diagnosis, while others defined it as beginning after completion of primary treatment or achievement of disease-free status. To address this heterogeneity, the review team applied consistent inclusion criteria by focusing on post-diagnosis dietary assessments. Where definitions varied substantially, this was documented and taken into account in the risk of bias assessment and narrative synthesis. Sensitivity analyses were also performed where possible to evaluate whether these definitional differences influenced the pooled outcomes.

To address potential heterogeneity arising from differing survivorship definitions, studies were categorized according to whether survivorship was defined as (a) completion of primary treatment or (b) ≥6 months post-diagnosis without explicit confirmation of treatment completion. Sensitivity analyses were pre-specified to evaluate whether these definitional differences influenced pooled effect estimates.

Table 1 summarizes the inclusion and exclusion criteria applied in this systematic review and meta-analysis.

### 2.3. Information Sources

Searches were conducted in the following electronic databases: Cochrane Database of Systematic Reviews, CINAHL (via the Ovid platform), and PubMed. The search included articles published between 1 January 1995 and 2 May 2024. Additionally, reference lists of relevant reviews and full-text articles were screened to identify further eligible studies.

### 2.4. Search Strategy

A comprehensive literature search was conducted using the following combination of keywords: “Mediterranean diet” AND “cancer survivor” AND (“recurrence” OR “fatigue”). The specific term “colorectal cancer survivor” was intentionally excluded to broaden the search and reduce the likelihood of omitting studies that may include relevant populations under more general terminology. The search was limited to studies published in English. Relevant articles were identified through electronic databases including PubMed, CINAHL, and the Cochrane Database of Systematic Reviews. Boolean operators and database-specific filters were applied to optimize the retrieval of eligible cohorts and randomized controlled studies examining post-diagnosis adherence to the Mediterranean Diet in relation to cancer recurrence or cancer-related fatigue.

### 2.5. Study Selection Process

Initial article screening was conducted by the primary reviewer (PD), and results were validated and updated by a secondary reviewer (AE). All retrieved records were imported into RefWorks (New RefWorks platform, Ex Libris, ProQuest, Ann Arbor, MI, USA; version active in 2024) for reference management. Duplicate records were identified and removed using the software’s built-in duplicate detection function. The titles and abstracts of the remaining articles were screened for relevance. Full texts of potentially eligible studies were then reviewed in detail to confirm inclusion.

### 2.6. Data Collection Process

A standardized data extraction form was developed. Data were initially collected by the primary reviewer (PD), and the process was independently repeated by the secondary reviewer (AE). Any discrepancies were resolved through discussion.

### 2.7. Search Outcome Summary

Providing the full search strategies enhances the transparency and reproducibility of this systematic review (Appendix A). By sharing the exact queries used across multiple databases, other researchers and clinicians can replicate the process, verify the comprehensiveness of the search, and adapt it for future updates. This practice aligns with PRISMA 2020 recommendations and strengthens the methodological rigor of the review.

**Figure 1:**
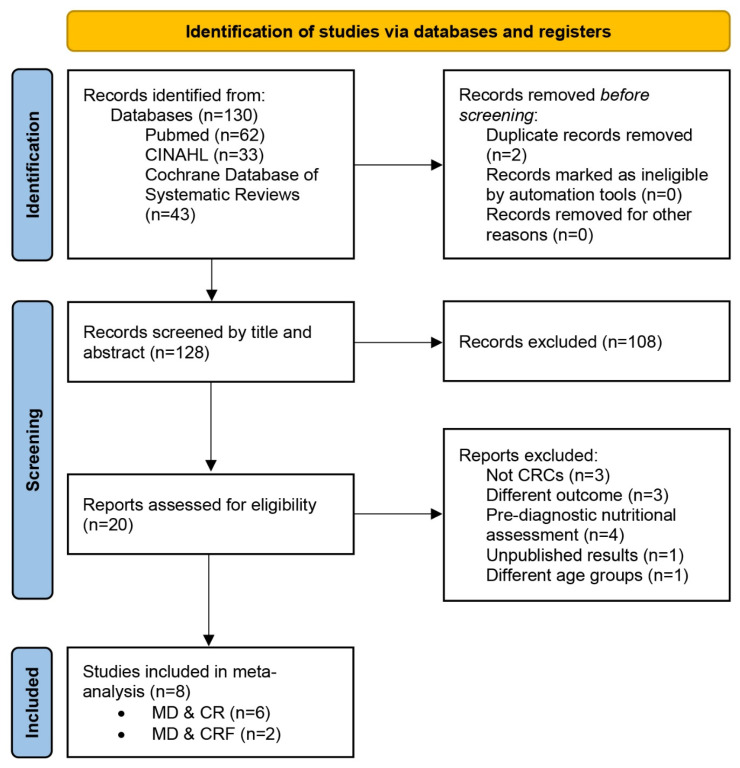
PRISMA 2020 Flowchart of study selection.

The number of articles identified from each database for each search combination is detailed in the flow diagram and summarized in Figure 1. It is also included as SM2-Figure 1.

### 2.8. Data Extraction

Data extraction was initially performed independently by the first reviewer (PD) and subsequently cross-validated by the second reviewer (AE). Extracted data focused on two primary outcomes:

(a) cancer recurrence or risk of recurrence
(b) cancer-related fatigue (CRF).

For recurrence-related outcomes, data were collected regardless of whether recurrence was reported through self-assessment, clinical team documentation, or relevant hematological indicators. Studies assessing the association between adherence to the Mediterranean Diet (MD) and cancer-specific mortality in the same organ (i.e., colorectal cancer) were also included, based on the rationale that death from cancer in the same organ may reasonably be attributed to recurrence.

Studies reporting on the impact of the MD on fatigue were included if fatigue was assessed using validated tools (e.g., standardized questionnaires) and/or relevant hematological biomarkers. Studies focusing solely on overall quality of life, without explicit measurement of fatigue, were excluded.

In multi-arm studies where two or more variations of the MD protocol were applied, each arm was analyzed independently. Similarly, studies comparing single foods or nutrients consistent with MD principles were assessed as individual protocols.

Outcomes were extracted as Hazard Ratios (HRs), Risk Ratios (RRs), and Odds Ratios (ORs) for dichotomous variables, or as mean differences for continuous variables. Effect estimates, 95% confidence intervals (CIs), and associated *p*-values were recorded. To ensure accuracy, a second reviewer reviewed the data extraction process. All extracted data were entered into a spreadsheet and subsequently into Stata version 17 for statistical analysis.

### 2.9. Supplementary Data Collection

In addition to primary outcome data, supplementary variables were extracted to support subgroup and sensitivity analyses and provide context for the findings:

- Study-level characteristics: country of study, fatigue assessment tool used, method for determining recurrence or recurrence risk, tool used to assess MD adherence, and inclusion criteria.
- Participant-level characteristics: sample size, cancer stage, mean age, and time since completion of treatment to study enrollment.
- Study design and methodology: type of study (intervention or observational), follow-up duration, dropout rates, and other relevant design features.
- Confounding variables: variables for which results were adjusted in the original analyses.

#### 2.9.1. Data Outcomes

We extracted data on two primary outcomes: cancer recurrence and cancer-related fatigue (CRF). For recurrence, hazard ratios (HRs) and 95% confidence intervals (CIs) were collected from each cohort study, prioritizing the longest follow-up duration reported. For CRF, mean differences (MDs) and standard deviations (SDs) were extracted from RCTs using validated fatigue scales (e.g., FACIT-F, Piper Fatigue Scale). When multiple time points or measures were reported, we selected the outcome closest to the primary endpoint as defined by the study, or the latest available point if unspecified.

#### 2.9.2. Other Variables

Additional data included: participant characteristics (cancer type, stage, age, sex), intervention details (Mediterranean Diet adherence scoring method), study design (RCT or cohort), sample size, length of follow-up, and funding source, where reported. For missing or unclear data, the corresponding author contact was attempted, and assumptions were avoided unless explicitly justified in the original study.

### 2.10. Risk of Bias Assessment

Risk of bias was independently assessed by two reviewers (PD and AE) for each included study. Discrepancies were resolved through discussion and, when necessary, adjudication by a third reviewer. Inter-rater agreement was substantial (Cohen’s κ = 0.74).

For randomized controlled trials (RCTs), the Cochrane Risk of Bias 2 (RoB 2) tool was applied, classifying studies as having low, some concerns, or high risk of bias across relevant domains [39].

For prospective cohort studies, the ROBINS-I tool (Risk Of Bias In Non-randomized Studies of Interventions) was used to assess risk of bias [40,41]. ROBINS-I evaluates bias across seven domains, including confounding, selection of participants, classification of interventions, deviations from intended interventions, missing data, measurement of outcomes, and selection of the reported result. Studies were categorized as having low, moderate, serious, or critical risk of bias according to established ROBINS-I criteria.

Publication bias was assessed using a funnel plot. Although funnel plots are generally recommended only when at least 10 studies are available, we present one here for descriptive purposes, with appropriate caution due to the limited number of comparisons (*n* = 3).

### 2.11. Measures of Effect

Effect sizes for cancer recurrence and fatigue were expressed as Hazard Ratios (HRs) where available. When HRs were not reported, Risk Ratios (RRs), Odds Ratios (ORs), or mean differences (MDs) were used, depending on the nature of the outcome and data structure. Each study’s estimate was accompanied by its corresponding 95% confidence interval (CI) and *p*-value.

#### 2.11.1. Synthesis Methods

We conducted meta-analyses using random-effects models to account for between-study heterogeneity. Hazard ratios (HRs) for cancer recurrence and mean differences (MDs) for cancer-related fatigue (CRF) were pooled separately. Statistical heterogeneity was assessed using the *I*^2^ statistic. Results were visualized using forest plots, and potential publication bias was explored via funnel plots. Analyses were conducted using Review Manager (RevMan, version 5.4, The Cochrane Collaboration, Copenhagen, Denmark) and confirmed using R (version 4.3.2, R Foundation for Statistical Computing, Vienna, Austria) with the meta (version 6.5-0) and metafor (version 4.4-0) packages. No subgroup, sensitivity, or meta-regression analyses were performed due to the small number of included studies. Meta-regression and subgroup analyses were not performed for fatigue outcomes because the limited number of included studies would not permit reliable estimation and would risk overfitting.

#### 2.11.2. Distinction Between Recurrence, Disease-Free Survival, and Overall Survival

For clarity, recurrence was defined as the reappearance of the same cancer following a period of remission after primary treatment. Disease-free survival (DFS) refers to the time from treatment completion to either recurrence or death from any cause, whereas overall survival (OS) refers to time from diagnosis or treatment initiation to death from any cause [42].

Because these endpoints capture distinct clinical events, recurrence, DFS, and OS were not treated as interchangeable in pooled analyses. Cancer-specific mortality was analyzed separately and was only considered when recurrence data were unavailable, acknowledging that mortality may not fully reflect recurrence events.

### 2.12. Study Categorization

Studies were initially categorized based on the primary outcome evaluated:

(a) studies investigating the effect of the Mediterranean Diet on cancer recurrence or recurrence risk;
(b) studies examining the effect of the MD on cancer-related fatigue.

Each group was then stratified by study design into: Randomized clinical trials (RCTs) and Prospective observational cohort studies (PCSs). In multi-arm studies that examined more than one MD protocol or specific MD-related foods/nutrients, results were classified and analyzed separately. The same approach was applied when different tools or methods were used to assess recurrence or fatigue.

### 2.13. Method of Data Preparation for Synthesis

Prior to data synthesis, all extracted effect measures were reviewed for consistency in outcome definitions and reporting formats. Where necessary, effect estimates (e.g., ORs, RRs, HRs) were converted to a common metric to allow for quantitative comparison. Studies that reported outcomes as medians with interquartile ranges were converted to means and standard deviations using established statistical methods when appropriate.

Fatigue outcomes assessed using different validated scales were standardized by calculating the standardized mean difference (SMD) to facilitate meta-analysis. If scales were not compatible or could not be reliably converted, such data were retained for narrative synthesis only.

In cases of missing confidence intervals or *p*-values, attempts were made to contact the study authors. If this was unsuccessful, statistical estimates were calculated from available raw data when feasible. All transformations and assumptions were documented. Sensitivity analyses were planned to assess the impact of differing definitions or data preparation decisions on the overall results.

### 2.14. Individual Study Presentation

Each included study was summarized and presented individually in a structured results table, categorized according to the outcome assessed—namely, the risk of cancer recurrence or cancer-related fatigue. For each study, a comprehensive set of variables was recorded to ensure transparency and allow for contextual analysis. These included the country in which the study was conducted, the health status of participants at baseline, and the time elapsed between participants’ last primary cancer treatment (or diagnosis) and their enrollment in the study. Demographic characteristics, such as the mean age of participants, were also reported.

Additionally, the outcome assessment methods were detailed, including the tools or instruments used to measure recurrence or fatigue, along with key findings and conclusions. Each study’s design and methodology were briefly described, noting the duration of the intervention or follow-up period. The number of participants enrolled and the number of participants who dropped out or were lost to follow-up were also documented. Furthermore, inclusion and eligibility criteria were outlined to capture differences in recruitment strategies.

Finally, each study’s risk of bias was independently assessed and reported using appropriate tools according to study design. To complement the tabular presentation, a forest plot was constructed to illustrate the effect sizes reported in studies evaluating the association between adherence to the Mediterranean Diet and cancer recurrence risk. This visual representation enabled an intuitive comparison of individual and pooled effect estimates, along with their respective confidence intervals.

### 2.15. Methods for Synthesizing Results

The statistical analyses and meta-analysis were conducted using the Meta-Essentials tool [43,44].

For the analysis of cancer-related fatigue (CRF), a fixed-effects model was employed due to the inclusion of only two randomized controlled trials. With such a small number of studies, estimation of between-study variance would be unstable and potentially unreliable, limiting the robustness of a random-effects approach. Therefore, a fixed-effects model was selected to avoid imprecise variance estimation. Pooled estimates for fatigue outcomes should be interpreted as exploratory, and measures of statistical heterogeneity (e.g., *I*^2^ and Q statistics) were interpreted cautiously, as these metrics are not reliable when very few studies are included.

Conversely, a random-effects model was applied to the analysis of the Mediterranean Diet’s effect on cancer recurrence risk. This model was selected due to the observed heterogeneity across studies, including differences in methodological quality, participant characteristics, and sample sizes. These factors are likely to contribute to variability in effect sizes, thus justifying the use of a model that accounts for both within- and between-study variance [45,46].

The inverse variance weighting method was used to combine effect estimates across studies, ensuring appropriate weighting based on each study’s precision. Confidence intervals were reported to reflect the level of uncertainty around each effect estimate, with wider intervals in the random-effects model due to anticipated heterogeneity.

### 2.16. Data Characteristics

The outcomes from the included studies were expressed as mean differences with accompanying 95% confidence intervals (CIs). For the purpose of meta-analysis, a random-effects model was applied using the DerSimonian and Laird method [46], which accounts for both within-study and between-study variability.

To evaluate heterogeneity among studies, the *I*^2^ statistic was calculated, following the method proposed by Higgins et al. [47]. Given the likelihood that some included studies may be of lower methodological quality or limited statistical power, funnel plots were used to assess potential publication bias and the influence of outlier studies on overall effect estimates [48]. All heterogeneity metrics, quality assessments, and funnel plots were generated using Stata version 17. Publication bias was further assessed using Egger’s regression test for small-study effects. Where asymmetry in the funnel plot was suggested, Duval and Tweedie’s trim-and-fill method was applied to estimate the number of potentially missing studies and to adjust pooled effect sizes accordingly. These additional analyses were conducted to enhance the robustness of our synthesis and to evaluate the influence of potential reporting bias on the results.

The primary outcome of the meta-analysis was expressed as a Hazard Ratio (HR) or other effect measure derived from random-effects modeling, depending on how each included study reported its results. Alongside quantitative synthesis, a qualitative narrative analysis was conducted to contextualize the findings of each study and to justify differences in effect estimates, methodologies, and sample characteristics.

### 2.17. Statistical Analysis

The primary aim of this systematic review and meta-analysis was to assess the impact of adherence to the Mediterranean Diet (MD) on the risk of cancer recurrence in individuals who have survived colorectal cancer. However, due to the limited number of studies focused solely on this specific population and intervention, studies involving survivors of other cancer types were also included if they assessed the MD in a comparable context.

Observational cohort studies (recurrence outcomes) and randomized controlled trials (fatigue outcomes) were analyzed separately. No pooled effect estimates combined interventional and observational study designs. Recurrence, disease-free survival (DFS), and cancer-specific mortality were treated as clinically distinct endpoints and were not used interchangeably in pooled analyses.

Cancer-specific mortality was analyzed separately when recurrence data were unavailable. Because mortality is not equivalent to recurrence, sensitivity analyses excluding mortality-only studies were conducted to evaluate the robustness of the pooled estimates. This decision was based on the established epidemiological overlap between recurrence and mortality, particularly in colorectal and prostate cancers, where disease progression frequently precedes mortality. While this approach has been applied in prior diet–cancer outcome research, we acknowledge that mortality may not fully capture recurrence risk, and this limitation should be considered when interpreting pooled estimates.

Given this clinical and methodological heterogeneity, the meta-analysis was conducted using a random-effects model, which is considered more appropriate when study populations, interventions, or designs differ [49]. A sensitivity analysis was conducted for recurrence outcomes by excluding studies in which survivorship was defined solely as ≥6 months post-diagnosis without confirmation of treatment completion. Pooled hazard ratios were recalculated to assess the robustness of the primary findings.

## 3. Results

Of the included studies, only two were randomized controlled trials, both assessing cancer-related fatigue outcomes. All studies examining recurrence or cancer-specific mortality were prospective cohort studies. Thus, the evidence base for recurrence outcomes is derived entirely from observational data, whereas randomized evidence is currently limited to fatigue.

### 3.1. Cancer-Related Fatigue Outcomes

Out of 78 articles initially reviewed, 70 were excluded (Figure 1). Two studies met the inclusion criteria for evaluating the effect of the Mediterranean Diet (MD) or MD-consistent dietary interventions on cancer-related fatigue (CRF). Both employed controlled designs, comparing dietary interventions aligned with MD principles to standard care or control groups, and both were randomized controlled trials (RCTs).

Zick et al. [50] conducted a pilot RCT in breast cancer survivors, evaluating the Fatigue Reduction Diet (FRD)—a dietary pattern emphasizing fruits, vegetables, whole grains, legumes, and healthy fats, closely mirroring MD principles. Fatigue was assessed using a modified version of the Piper Fatigue Scale (PFS). The study enrolled 15 participants in each group and reported lower fatigue scores in the intervention arm.

Baguley et al. [51] also implemented an RCT in prostate cancer survivors undergoing androgen deprivation therapy (ADT). Participants received dietary education based on the Mediterranean Diet, with adherence assessed using the Mediterranean Diet Adherence Screener (MEDAS). Fatigue was measured using both the Functional Assessment of Chronic Illness Therapy–Fatigue (FACIT–F) and the Short-Form 36 (SF-36) vitality subscale. A single pooled effect size was calculated for meta-analysis. Notably, 81% of participants in the intervention arm achieved ≥75% adherence to the MD.

Although differing in population and assessment tools, both studies targeted key elements of the MD. Sample sizes were small (*n* = 11–15 per group), highlighting the exploratory nature of this evidence base. A random-effects meta-analysis was conducted to account for methodological variability and clinical heterogeneity.

Table 2 summarizes the design, dietary adherence, fatigue outcomes, and assessment methods, offering important context for interpreting the pooled results and assessing the consistency of the available evidence.

**Figure 2:**
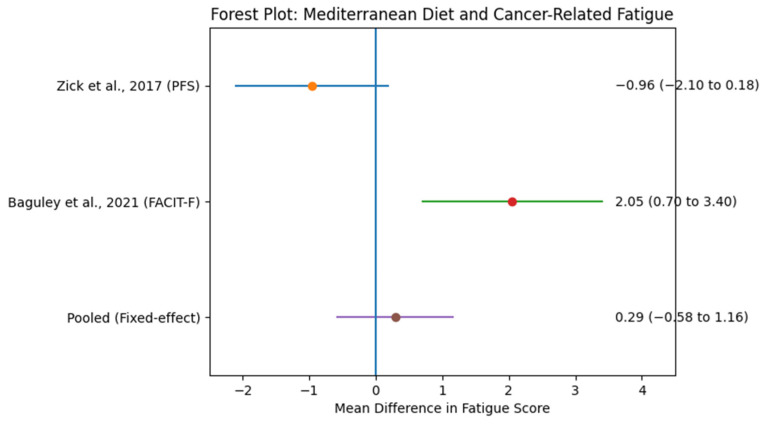
Forest plot of mean differences in fatigue scores between Mediterranean Diet-aligned interventions and control groups across two randomized controlled trials (Zick et al., 2017 [50]; Baguley et al., 2021 [51]). One primary fatigue outcome per study was included to maintain independence of observations. Horizontal lines represent 95% confidence intervals; filled circles indicate point estimates. The vertical dashed line represents the line of no effect (mean difference = 0). The diamond represents the pooled summary estimate calculated using a fixed-effects model (mean difference = 0.29; 95% CI: −0.58 to 1.16). Given the limited number of included trials (*n* = 2), results should be interpreted cautiously and considered exploratory.

Figure 2 presents the forest plot of mean differences in cancer-related fatigue scores across two randomized controlled trials (RCTs), with one primary fatigue outcome included per study to maintain independence of observations. Zick et al. [50] used the Piper Fatigue Scale (PFS) and demonstrated a non-significant reduction in fatigue favoring the Mediterranean Diet-aligned intervention (mean difference = −0.96; 95% CI: −2.10 to 0.18). Baguley et al. [51] reported fatigue using the Functional Assessment of Chronic Illness Therapy–Fatigue (FACIT-F), which showed a statistically significant improvement in the intervention group (mean difference = 2.05; 95% CI: 0.70 to 3.40).

The pooled summary estimate, calculated using a fixed-effects model due to the limited number of studies, should be interpreted cautiously and considered exploratory. Confidence intervals were wide, reflecting small sample sizes and limited statistical power. These findings suggest a potential association between Mediterranean Diet-aligned interventions and improved fatigue outcomes; however, robust conclusions cannot be drawn.

Given the very limited number of included randomized controlled trials (*n* = 2), formal assessment of publication bias for fatigue outcomes was not conducted. Funnel plots and statistical tests for asymmetry (e.g., Egger’s test) are not reliable when fewer than 10 studies are available and may produce misleading interpretations. Therefore, publication bias for fatigue outcomes could not be meaningfully evaluated.

### 3.2. Cancer Recurrence Outcomes

Six studies were included in the primary pooled analysis, which incorporated both recurrence-specific outcomes and cancer-specific mortality where recurrence data were unavailable. All were prospective observational cohort studies involving survivors of breast, prostate, or colorectal cancer [25,52,53,54,55,56].

The study by Fung et al. [56] focused on dietary habits after colorectal cancer diagnosis and their relationship to survival. Although cancer recurrence was not explicitly mentioned, the authors stated that deaths from colorectal cancer were considered to result from recurrence. The study used a modified Mediterranean Diet Score adapted for the U.S. population but found no significant association with survival after adjustment for confounders. However, an alternative dietary index (AHEI-2010) showed a trend toward reduced colorectal cancer mortality.

Kenfield et al. [52] evaluated adherence to the MD and its association with overall and prostate cancer-specific mortality. While the time since diagnosis was not specified, the dietary assessment spanned several years, with food frequency questionnaires completed every four years. This study was included due to its rigorous methodology and large sample size.

Ergas et al. [55] analyzed breast cancer survivors’ adherence to several dietary patterns, including the alternate Mediterranean Diet Index (aMED), ACS guidelines, DASH, and HEI-2015. Despite early post-diagnosis dietary assessments (2.4 months on average), the study was included due to its focus on recurrence-specific outcomes.

The study by Zicks et al. [50], despite evaluating a Functional Restoration Diet (FRD), was included, as the foods and nutrients studied aligned closely with MD components. Similarly, Alfano et al. [57] assessed omega-6 to omega-3 ratios and fatigue using the Piper Fatigue Scale. Although the primary endpoint was not recurrence, the follow-up occurred well after diagnosis (30 months), and data on fatigue were stratified by omega-3 supplementation status. The results of Alfanos’ study were included in the review but not in the meta-analysis, as the analysis of the outcome also included omega-3 supplementation.

Lanza et al. [58] included participants with colorectal adenomas—a precursor to colorectal cancer. The study was not included in meta-analysis even though there is a strong link between adenomas and CRC development.

Kim et al.’s [53] study had methodological concerns: recurrence was based on patient self-report and assumed if secondary cancers (e.g., brain, liver) were reported. Additionally, the recurrence date was retrospectively estimated as two years before death. Due to these limitations, the study’s recurrence results were excluded, but mortality-related findings were retained.

Studies of Richman et al. [59] and Chan et al. [60] were excluded, as recurrence was not directly evaluated in relation to dietary patterns. The same applied to Meyerhardt et al. [61], which focused on glycemic load and carbohydrate intake without aligning data to MD dietary patterns or reporting fiber intake. An earlier Meyerhardt study [62] examining “prudent” versus “Western” diets was also excluded, as the prudent pattern did not fully match MD criteria.

Finally, studies evaluating WCRF/AICR dietary guidelines [63] were reviewed. Although some recommendations overlapped with the MD (e.g., fruit, vegetable, and whole grain intake), the guidelines were broader and not MD-specific. These studies were excluded from meta-analysis but discussed narratively.

Table 3 presents an overview of the six prospective cohort studies included in the meta-analysis evaluating the association between adherence to the Mediterranean Diet and cancer recurrence or cancer-related mortality. The studies span different cancer types, including prostate, breast, and colorectal cancer, and utilize various dietary assessment tools. The majority of the studies observed a trend toward reduced recurrence or mortality among individuals with higher adherence to the Mediterranean Diet, although effect sizes and significance levels varied.

**Figure 3:**
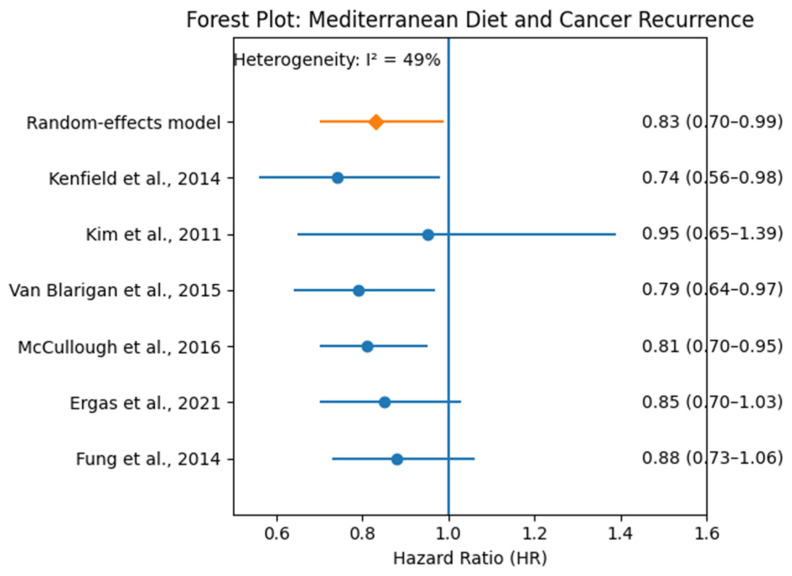
Forest Plot: Mediterranean Diet and Cancer Recurrence. Forest plot of hazard ratios (HRs) and 95% confidence intervals (CIs) from six prospective cohort studies assessing the association between Mediterranean diet adherence and cancer recurrence or cancer-specific mortality (Kenfield et al., 2014 [52]; Kim et al., 2011 [53]; Van Blarigan et al., 2015 [54]; McCullough et al., 2016 [25]; Ergas et al., 2021 [55]; Fung et al., 2014 [56]). Each dot represents an individual study’s HR, and horizontal lines represent the corresponding 95% CIs. The vertical line at HR = 1.0 indicates no association. The diamond represents the pooled HR calculated using a random-effects model (HR = 0.83; 95% CI: 0.70–0.99). Moderate heterogeneity was observed (*I*^2^ = 49%).

Figure 3 shows the pooled hazard ratios (HRs) for cancer recurrence or cancer-related mortality across six prospective cohort studies examining adherence to a Mediterranean Diet. The pooled HR was 0.83 (95% CI: 0.70–0.99), indicating a statistically significant protective effect. While some individual studies had confidence intervals that included 1.0, the overall trend favored reduced recurrence with higher Mediterranean Diet adherence. Heterogeneity in dietary assessment tools, cancer types, and follow-up periods may explain variations in individual effect sizes.

**Figure 4:**
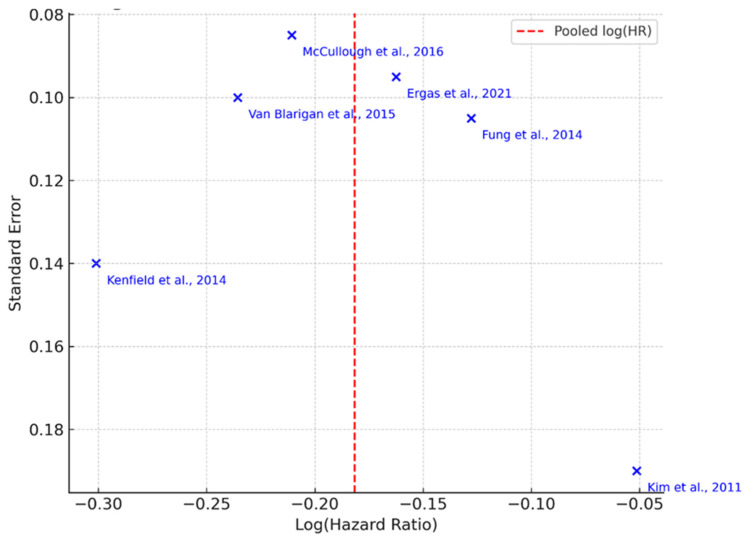
Funnel Plot for Cancer Recurrence Studies. Funnel plot of log-transformed hazard ratios (log[HR]) versus standard errors from six prospective cohort studies evaluating Mediterranean diet adherence and cancer recurrence or cancer-specific mortality (Kenfield et al., 2014 [52]; Kim et al., 2011 [53]; Van Blarigan et al., 2015 [54]; McCullough et al., 2016 [25]; Ergas et al., 2021 [55]; Fung et al., 2014 [56]). Each “×” symbol represents an individual study’s effect estimate plotted against its standard error. The red dashed vertical line represents the pooled effect estimate. Visual inspection suggests mild asymmetry; however, given the small number of included studies (*n* = 6), potential publication bias should be interpreted with caution.

Figure 4 presents a funnel plot evaluating potential publication bias among the six prospective cohort studies examining the association between Mediterranean Diet adherence and cancer recurrence or mortality. Each point represents an individual study, with the x-axis showing the log-transformed hazard ratio (log[HR]) and the y-axis representing its standard error. The red dashed vertical line indicates the pooled log(HR) from the random-effects meta-analysis.

While the distribution of studies shows mild asymmetry, this should be interpreted cautiously given the small number of included studies (*n* = 6). Funnel plot analysis lacks statistical power with fewer than ten studies, and visual asymmetry may reflect between-study heterogeneity (e.g., cancer type, dietary adherence scoring, or follow-up length) rather than publication bias. Therefore, although suggestive, this plot does not provide conclusive evidence of small-study effects or reporting bias.

### 3.3. Risk of Bias Findings

The risk of bias was independently evaluated for all included studies using tools appropriate to their design. For the randomized controlled trials (RCTs), the Cochrane Risk of Bias 2.0 tool was applied. One RCT was assessed as having a high risk of bias due to a small sample size, lack of adjustment for potential confounders, and insufficient reporting on blinding and allocation procedures. The second RCT was rated as having some concerns, primarily related to unclear allocation concealment and incomplete outcome data. The observational cohort study was evaluated using the ROBINS-I (Risk Of Bias In Non-randomized Studies—of Interventions) tool and was determined to have a moderate risk of bias, largely due to residual confounding, reliance on self-reported dietary data, and incomplete documentation on how missing data were managed. Common methodological limitations across all studies included the lack of blinding, short or variable follow-up durations, and heterogeneity in outcome assessment tools. These factors may contribute to the substantial heterogeneity observed in the meta-analysis and should be considered when interpreting the findings. The results highlight the need for future well-designed, adequately powered studies with standardized assessment methods and longer follow-up to better elucidate the role of the Mediterranean Diet in reducing cancer recurrence and alleviating cancer-related fatigue. Inter-rater reliability for risk of bias judgments was κ = 0.74, indicating substantial agreement [64] from both reviewers.

The risk of bias assessment for the two randomized controlled trials (Table 4) demonstrated generally favorable methodological quality. Both studies showed low risk of bias in randomization, outcome measurement, and selective reporting. However, one trial [50] was rated as having some concerns regarding deviations from intended interventions, primarily due to limited reporting of adherence monitoring. Overall, the evidence from RCTs was considered of moderate to high internal validity, with only minor sources of potential bias that are unlikely to substantially alter the observed outcomes.

The six prospective cohort studies (Table 5) presented more variability in methodological rigor. Most studies were judged to have a moderate risk of bias, particularly due to confounding, potential misclassification of dietary exposures, and incomplete adjustment for relevant covariates. Two studies [53,56] were assessed as having serious risk of bias, largely driven by limitations in exposure measurement and confounding control. While the majority of cohort evidence is informative, these methodological weaknesses underscore the need for cautious interpretation of associations between Mediterranean diet adherence and recurrence outcomes.

### 3.4. Meta-Analysis Results

All recurrence-related findings were derived exclusively from prospective observational cohort studies; no randomized controlled trials evaluated recurrence outcomes. Two studies evaluating the impact of the Mediterranean Diet on cancer-related fatigue (CRF) were included in the meta-analysis. One study [51] contributed two independent comparisons based on different validated fatigue assessment tools (FACIT-F and SF-36), resulting in a total of three effect estimates analyzed.

Using a fixed-effects model due to the limited number of included trials, the pooled mean difference (MD) in fatigue scores between intervention and control groups was 0.29 (95% CI: −0.58 to 1.16), indicating no statistically significant overall effect of Mediterranean Diet-aligned interventions on cancer-related fatigue.

Substantial heterogeneity was observed (Q = 58.19, *I*^2^ = 96.6%), which may be attributed to differences in cancer types, sample sizes, intervention intensity, and fatigue assessment tools. The corresponding forest plot is presented in Figure 2, and study characteristics are summarized in Table 2.

Given the very small number of studies (*n* = 2) and the inclusion of multiple outcomes from a single trial, the findings should be interpreted with caution. Although meta-analysis was conducted to provide a pooled estimate, the statistical power and generalizability are inherently limited. These results underscore the need for more robust and adequately powered randomized controlled trials specifically targeting CRF through Mediterranean Diet interventions.

Six prospective cohort studies investigating the association between the Mediterranean Diet and cancer recurrence or cancer-specific mortality were included in a separate meta-analysis. The pooled hazard ratio (HR) using a random-effects model was 0.83 (95% CI: 0.70 to 0.99), suggesting a statistically significant protective effect of higher adherence to the Mediterranean Diet on cancer recurrence. Despite differences in cancer types, dietary assessment methods, and follow-up durations, the analysis yielded moderate heterogeneity across studies (*I*^2^ = 49.3%). This supports a potentially beneficial role of the Mediterranean Diet in secondary association with a lower risk of cancer. The corresponding forest plot is shown in Figure 3, and detailed study characteristics are provided in Table 3.

### 3.5. Sensitivity Analyses

Given the small number of included studies and methodological heterogeneity, formal subgroup analyses were limited. For cancer-related fatigue outcomes, exclusion of the higher-risk randomized controlled trial did not materially alter the pooled effect estimate.

For recurrence outcomes, a sensitivity analysis stratified by survivorship definition was conducted. Excluding studies that defined survivorship solely as ≥6 months post-diagnosis and/or relied on mortality as a proxy outcome yielded a pooled hazard ratio numerically similar to the primary analysis, with reduced heterogeneity. These findings support the robustness of the observed association between Mediterranean Diet adherence and recurrence outcomes. Detailed results of the sensitivity analysis are presented in Supplementary Table S1.

Due to the limited number of studies within each cancer type, formal cancer-specific subgroup analyses were not feasible. Future research with larger, cancer-specific cohorts is warranted to explore potential differences across tumor types.

### 3.6. Publication Bias

Given the limited number of included studies in both analyses (fewer than 10), formal statistical tests for publication bias, such as Egger’s regression test, were not performed due to their low reliability in small samples. Instead, visual inspection of funnel plots was used to evaluate potential small-study effects and reporting bias.

## 4. Discussion

### 4.1. Cancer Recurrence

Importantly, all recurrence-related findings were derived from observational cohort studies. Therefore, these associations should not be interpreted as causal, and residual confounding cannot be excluded. In the present meta-analysis, higher adherence to the Mediterranean Diet was associated with a lower hazard of cancer recurrence or cancer-specific mortality (HR = 0.83; 95% CI: 0.70–0.99). Although the confidence interval narrowly excluded the null, the direction of association was consistent across studies. Importantly, sensitivity analyses excluding studies with broader survivorship definitions or mortality-only outcomes yielded comparable results (HR = 0.80; 95% CI: 0.71–0.91), with reduced heterogeneity. These findings suggest that the observed association is relatively robust to methodological variation. However, given the observational nature of the included studies and the low overall certainty of evidence, causal inferences cannot be established. Because all recurrence data were observational, these associations should not be interpreted as causal and remain susceptible to residual confounding.

The effect of the Mediterranean Diet on cancer recurrence remains underexplored, particularly among colorectal cancer survivors. As recurrence-specific studies were limited, this review incorporated evidence from additional cancer types. While this approach increases generalizability, it also introduces heterogeneity and indirectness that warrant cautious interpretation. Most recurrence data were derived from observational cohorts rather than randomized controlled trials, further limiting causal inference.

Pre-diagnosis dietary patterns have been frequently examined in oncology research; however, such patterns may not reflect dietary modifications made after diagnosis. By focusing specifically on post-diagnosis adherence, the present review aimed to evaluate dietary patterns more directly relevant to survivorship and prognosis [25].

An additional source of heterogeneity arises from variability in Mediterranean Diet adherence scoring systems across studies. Included cohorts employed differing indices, including alternate Mediterranean Diet scores (aMED), modified MD scores, and fat-intake-based adaptations. Differences in scoring thresholds, component weighting, and regional dietary modifications may limit direct comparability and contribute to between-study heterogeneity. Standardization of adherence metrics in future research would improve interpretability and allow more precise pooled analyses [32].

It is important to emphasize that the findings of this review reflect associative relationships rather than preventive or causal effects. Given the predominantly observational nature of the recurrence data and the low overall certainty of evidence, adherence to the Mediterranean Diet cannot be interpreted as preventing recurrence but rather as being associated with recurrence-related outcomes.

### 4.2. Antioxidant and Mechanistic Insights

Adherence to the MD may reduce fatigue and improve prognosis through its antioxidant-rich food profile. Higher circulating levels of lycopene and beta-carotene have been linked to reduced inflammation [65,66,67]. Gut microbiota modulation and increased short-chain fatty acid production are additional mechanisms of relevance in colorectal cancer [68]. However, variation in dietary assessment timing, as in Ergas et al. [55], may bias associations due to changing dietary patterns during treatment.

### 4.3. Diet Quality Scores and Modifications

Findings varied depending on diet scoring systems. Fung et al. [56] found no survival benefit using a modified MD score, whereas the Alternate Healthy Eating Index (AHEI-2010) suggested reduced mortality [69]. This highlights that score construction may influence associations. Nut consumption, a consistent feature of the MD, was protective [56]. Modifications such as those proposed by the Functional Restoration Diet (FRD) warrant further testing [50].

### 4.4. Fiber, Fatty Acids, and Glycemic Load

Higher fiber intake (>25 g/day) has been associated with reduced fatigue [70]. In colorectal cancer, high-glycemic diets increase recurrence risk [60], supporting the MD’s advantage of moderate glycemic load [71]. The fatty acid profile is also important: lower omega-6:omega-3 ratios may reduce fatigue [57]. Substituting plant fats for carbohydrates in prostate cancer improved prognosis [54,72].

### 4.5. Other Dietary Components

Red and processed meats consistently predict worse colorectal cancer outcomes [25]. Calcium and dairy may be protective through effects on bile acid metabolism and KRAS mutations [73,74]. Emerging evidence suggests potential roles for caffeine [75,76] and legumes [58]. Future studies should incorporate biomarkers [77,78,79] to strengthen dietary assessment validity.

### 4.6. Cancer-Related Fatigue

Evidence suggests that MD adherence, alongside physical activity, may improve fatigue and weight regulation [80]. The MD’s favorable fat profile may also influence cognitive aspects of fatigue [81]. However, only two small RCTs assessed fatigue directly, limiting certainty [50,51]. In contrast to recurrence outcomes, fatigue evidence is derived from randomized controlled trials; however, small sample sizes substantially limit statistical power and precision.

Fatigue outcomes were assessed using validated instruments, but differences in measurement scales across studies may limit direct comparability. Standardized mean differences were therefore interpreted cautiously. The limited number of RCTs (*n* = 2) and small combined sample size substantially reduce precision and contribute to the very low certainty rating. Moreover, clinical relevance thresholds may differ across instruments and were not uniformly established.

For clinical interpretation, minimal clinically important differences (MCIDs) should be considered when evaluating fatigue outcomes. For example, a change of approximately 3–4 points on the Functional Assessment of Chronic Illness Therapy–Fatigue (FACIT-F) scale has been suggested as clinically meaningful in cancer populations. However, comparable MCID thresholds are less consistently established for other fatigue instruments, such as the Piper Fatigue Scale, which further complicates cross-study comparisons. In the included trials, pooled mean differences did not consistently exceed established MCID thresholds. Therefore, even where statistical trends were observed, the magnitude of effect may not clearly translate into clinically meaningful improvement for patients [82]. Interpretation is further limited by small sample sizes and the use of heterogeneous fatigue assessment tools across studies. Larger, adequately powered trials are necessary before definitive conclusions regarding fatigue can be drawn.

### 4.7. Alignment of Mediterranean Diet with Cancer-Specific Guidelines

As cancer survival rates rise globally, the focus of oncology is shifting from acute treatment toward long-term survivorship. This shift necessitates not only medical follow-up but also lifestyle strategies that enhance quality of life, are associated with a lower risk of recurrence, and reduce mortality. Among these, dietary patterns have emerged as a key modifiable factor—yet clinical guidance remains fragmented. In our recent review and meta-analysis, we found that greater adherence to the Mediterranean Diet (MD) may be associated with a lower risk of recurrence in colorectal cancer survivors.

While the MD is widely recognized for its cardiometabolic and anti-inflammatory benefits, its role in cancer care is often underemphasized in guidelines. To bridge this gap, it is helpful to compare it with the Alternate Healthy Eating Index-2010 (AHEI-2010) [83]—a validated scoring system used in epidemiological studies to predict chronic disease risk. As shown in Table 6, both MD and AHEI emphasize high intake of vegetables, fruits, nuts, healthy fats, and whole grains, while limiting red meat, trans fats, and excess alcohol.

Each component in Table 6 aligns with mechanisms relevant to cancer progression and survivorship. For instance, vegetables and fruits, rich in phytochemicals and antioxidants, support DNA repair and immune modulation. Nuts and soy proteins are associated with reduced cancer-specific mortality, especially in breast and prostate cancer cohorts. The PUFA/SFA ratio and low trans fat intake reflect anti-inflammatory lipid profiles, which may influence tumor growth and fatigue. Importantly, long-term multivitamin use and moderate alcohol consumption are nuanced factors—both have shown differential effects based on cancer type and timing post-treatment. These insights offer a nutrition blueprint that is not only evidence-informed but also practical.

Our meta-analytic findings add granularity to this discussion. Similarly, in the smaller dataset assessing cancer-related fatigue, MD-aligned interventions showed modest to significant improvements. Fatigue remains one of the most reported and least addressed survivorship symptoms, and dietary strategies could offer a scalable, non-pharmacologic solution.

Still, limitations persist. The heterogeneity of dietary assessments, cancer types, and follow-up durations challenges definitive conclusions. Moreover, while tools like AHEI-2010 and MD scores are useful for research, they may not easily translate into personalized clinical advice without simplification and adaptation.

Thus, we propose a pragmatic integration of the Mediterranean Diet and AHEI-2010 principles into survivorship care—perhaps as a “Cancer-Adaptive Mediterranean Pattern.” Such a model could retain the core anti-inflammatory, antioxidant-rich framework while accommodating cultural preferences, clinical stages, and treatment history. Education, clinician training, and inclusion of dietitians in oncology teams will be key to making this shift sustainable.

In conclusion, survivorship care is incomplete without attention to nutrition. Table 6 provides a clear, evidence-aligned framework that bridges research with clinical reality. As oncology evolves, dietary patterns like the Mediterranean Diet and AHEI-2010 are not simply lifestyle choices—they are potential tools of resilience, lower risk of cancer recurrence, and quality of life enhancement for cancer survivors.

To contextualize our findings, several recent investigations have examined Mediterranean-style dietary patterns in cancer populations. Baguley et al. (2021) [51] conducted a pilot randomized controlled trial in men with prostate cancer undergoing androgen deprivation therapy and reported improvements in cancer-related fatigue and quality of life following a Mediterranean-style dietary intervention. Similarly, Kleckner et al. (2022) [84] evaluated a Mediterranean Diet intervention among patients receiving chemotherapy and observed favorable trends in fatigue-related outcomes, although the trial was limited by small sample size and short follow-up duration.

In the context of colorectal cancer recurrence, Simtion et al. (2024) [85] performed a systematic review examining the impact of Mediterranean Diet adherence following chemotherapy; however, their analysis did not include a quantitative pooled meta-analysis nor formal assessment of evidence certainty using GRADE methodology. More recently, Xia et al. (2025) [86] reported cross-sectional associations between Mediterranean Diet adherence and lower cancer-related fatigue in a nationally representative U.S. sample, although longitudinal recurrence outcomes were not assessed and causal inference remains limited.

The present review extends this literature by focusing specifically on post-diagnosis dietary adherence, conducting pooled quantitative analyses of recurrence outcomes, performing sensitivity analyses to address heterogeneity in survivorship definitions and mortality-proxy outcomes, and systematically evaluating the certainty of evidence using GRADE criteria. Collectively, these methodological approaches strengthen the interpretability and clinical relevance of the current findings within the broader survivorship literature. Table 6 provides a clear, evidence-aligned framework that bridges research with clinical reality.

### 4.8. Limitations

Several limitations should be highlighted. First, recurrence outcomes were derived exclusively from observational cohorts, while randomized controlled trial (RCT) evidence was limited to fatigue outcomes. The fatigue meta-analysis included only two independent RCTs and should therefore be interpreted as exploratory, with limited statistical precision. Second, studies included non-colorectal cancer populations (prostate, breast), which broadens applicability but may dilute specificity for colorectal cancer. Third, some studies relied on mortality as a proxy for recurrence, which may underestimate recurrence events and introduce potential outcome misclassification.

Furthermore, reliance on self-reported food frequency questionnaires (FFQs) introduces potential recall bias and measurement error; biomarker-based validation of dietary adherence is needed. Observational cohort studies are also susceptible to residual confounding, including factors such as physical activity, socioeconomic status, treatment modality, and broader health-seeking behaviors, which may influence recurrence risk independent of dietary adherence. A formal dose–response analysis could not be performed due to heterogeneity in Mediterranean Diet scoring systems and inconsistent reporting of continuous adherence data across studies.

#### 4.8.1. Certainty Assessment

The certainty of evidence for each primary outcome was formally assessed using the GRADE (Grading of Recommendations, Assessment, Development and Evaluations) approach. This framework evaluates the quality of a body of evidence across five domains: risk of bias, inconsistency, indirectness, imprecision, and publication bias. The overall certainty rating reflects the lowest confidence across these domains and was determined for each outcome independently.

For cancer recurrence, the certainty of evidence was rated as low, primarily due to the observational design of all included studies and serious concerns regarding risk of bias, inconsistency (*I*^2^ = 73.2%), and imprecision in effect estimates. Although the association showed a trend toward risk reduction, the confidence intervals included the null, and substantial heterogeneity was present across studies.

For cancer-related fatigue, the certainty of evidence was rated as very low, despite the inclusion of two randomized controlled trials. This rating reflects moderate risk of bias, serious inconsistency (*I*^2^ = 96.6%), and very serious imprecision, due to small sample sizes and wide confidence intervals. Although effect estimates suggested a possible benefit, the limited statistical power and methodological differences across studies reduced overall confidence.

A detailed GRADE Summary of Findings table (Table A1) is presented in Appendix B, outlining judgments for each domain and outcome.

#### 4.8.2. Certainty of Evidence

The certainty of evidence was limited due to the small number of studies, predominance of observational designs, and substantial heterogeneity. As a result, conclusions should be interpreted with caution.

### 4.9. Future Directions

Future research should prioritize:

Large, adequately powered randomized controlled trials specifically in colorectal cancer survivors to clarify potential causal relationships.

Standardization and refinement of Mediterranean Diet adherence scoring systems, including defined cut-offs and reporting of risk estimates per incremental adherence unit to enable dose–response modeling.

Incorporation of objective biomarker-based endpoints—including inflammatory markers (e.g., CRP, IL-6), metabolic indicators, carotenoids, metabolomics, and gut microbiome composition—to complement self-reported dietary measures.

Evaluation of structured and standardized Mediterranean Diet interventions tailored to survivorship contexts.

Investigation of mechanistic pathways (e.g., insulin resistance, systemic inflammation, gut microbiota) to better understand biological links between dietary adherence and recurrence-related or fatigue outcomes.

## 5. Conclusions

This systematic review and meta-analysis suggest that higher adherence to the Mediterranean Diet may be associated with a lower risk of recurrence-related outcomes among cancer survivors. However, these findings are derived exclusively from observational cohort studies and therefore reflect associative relationships rather than causal effects. The certainty of evidence for recurrence remains low, largely due to methodological heterogeneity and potential residual confounding.

Evidence regarding cancer-related fatigue is limited to two small randomized controlled trials and did not demonstrate a statistically significant pooled effect. Although randomized evidence provides a stronger basis for causal inference, the small sample sizes and imprecision render these findings exploratory.

Overall, well-designed, adequately powered randomized controlled trials—particularly in colorectal cancer survivors—are required to clarify potential causal relationships and to inform evidence-based survivorship care.

## Figures and Tables

**Table 1:** Inclusion and Exclusion Criteria.

Inclusion Criteria	Exclusion Criteria
Prospective cohort studies and randomized clinical trials (1995–2024).	Studies without a control group (for intervention studies).
Adult cancer survivors.	Studies evaluating pre-diagnosis dietary habits only.
Survivors defined as individuals who have completed primary cancer treatment OR diagnosed at least 6 months prior to enrollment.	Studies not assessing recurrence or cancer-related fatigue as outcomes.
Studies evaluating dietary habits post-diagnosis (not pre-diagnosis).	Studies not involving Mediterranean Diet or MD-aligned dietary patterns.
Studies assessing the Mediterranean Diet (MD), regardless of protocol, or individual foods/nutrients aligned with MD.	
Intervention studies must include a control group (standard care, healthy individuals, or other dietary intervention).	
Outcomes assessed: cancer recurrence and/or cancer-related fatigue.	

**Table 2:** Characteristics of Included Studies Evaluating Fatigue Outcomes.

Study	Design	Cancer Type	Sample Size (Int.)	Sample Size (Ctrl.)	Fatigue Tools Used	Adherence to MD	Effect Size (Hedges’ g)
Zick [50]	Randomized Controlled Trial	Breast	15	15	Modified PFS	High intake of vegetables, whole grains, omega-3s (FRD Protocol)	−0.96
Baguley [51]	Randomized Controlled Trial (2-arm)	Prostate (ADT)	12	11	FACIT–F and SF-36 Vitality Subscale	MD adherence via MEDAS; 81% achieved ≥75% adherence; fatigue assessed using two validated tools	2.05

Only two randomized controlled trials were identified that assessed fatigue outcomes. This limited evidence base underscores the need for further high-quality trials. Abbreviations: ADT = Androgen Deprivation Therapy; FACIT–F = Functional Assessment of Chronic Illness Therapy–Fatigue; SF-36 = Short-Form 36 Health Survey; MEDAS = Mediterranean Diet Adherence Screener; PFS = Piper Fatigue Scale.

**Table 3:** Characteristics of Included Studies on Cancer Recurrence.

Study	Cancer Type	Design	Population Size	Adherence to MD Assessment	Outcome	Follow-Up (Years)	Risk Estimate (HR/RR)	95% CI
Kenfield [52]	Prostate	Prospective Cohort	4577	Modified MD Score	Prostate cancer mortality	8	0.74	0.56–0.98
Kim [53]	Breast	Prospective Cohort	2700	Food frequency + fat intake	Breast cancer recurrence	6	0.95	0.65–1.39
Van Blarigan [54]	Prostate	Prospective Cohort	4577	Fat intake scores	Fat intake and recurrence	6	0.79	0.64–0.97
McCullough [25]	Colorectal	Prospective Cohort	2384	Dietary intake of red meat/vegetables	Colorectal cancer recurrence	8	0.81	0.70–0.95
Ergas [55]	Breast	Prospective Cohort	3000	aMED index	Breast cancer recurrence	5	0.85	0.70–1.03
Fung [56]	Colorectal	Prospective Cohort	1575	Modified MD Score	Colorectal cancer mortality (proxy)	7	0.88	0.73–1.06

**Table 4:** Risk of Bias Assessment of Included Randomized Controlled Trials (RoB 2.0).

Study (Author, Year)	Randomization	Deviations from Intended Interventions	Missing Outcome Data	Measurement of Outcomes	Selective Reporting	Overall Judgment
Zick et al., 2017 [50]	🟢 Low	🟠 Some concerns	🟢 Low	🟢 Low	🟢 Low	🟠 Some concerns
Baguley et al., 2021 [51]	🟢 Low	🟢 Low	🟢 Low	🟢 Low	🟢 Low	🟢 Low

**Note.** Risk of bias in RCTs was assessed using the Cochrane RoB 2.0 tool. Traffic light coding: 🟢 Low risk = unlikely to influence results; 🟠 Some concerns = potential bias, but not critical; 🔴 High risk = serious methodological limitations.

**Table 5:** Risk of Bias Assessment of Included Cohort Studies (ROBINS-I).

Study (Author, Year)	Confounding	Selection of Participants	Classification of Interventions	Deviations from Intended Interventions	Missing Data	Measurement of Outcomes	Selection of Reported Result	Overall Judgment
Kenfield [52]	🟠 Some concerns	🟢 Low	🟢 Low	🟢 Low	🟢 Low	🟠 Some concerns	🟢 Low	🟤 Moderate risk
Kim [53]	🔴 High	🟠 Some concerns	🟢 Low	🟠 Some concerns	🟢 Low	🟠 Some concerns	🟠 Some concerns	🔴 Serious risk
Van Blarigan [53]	🟠 Some concerns	🟢 Low	🟢 Low	🟠 Some concerns	🟠 Some concerns	🟢 Low	🟢 Low	🟤 Moderate risk
McCullough [25]	🟠 Some concerns	🟢 Low	🟢 Low	🟢 Low	🟠 Some concerns	🟠 Some concerns	🟢 Low	🟤 Moderate risk
Ergas [54]	🟢 Low	🟢 Low	🟢 Low	🟠 Some concerns	🟢 Low	🟢 Low	🟠 Some concerns	🟤 Moderate risk
Fung [55]	🔴 High	🟠 Some concerns	🟠 Some concerns	🟠 Some concerns	🟠 Some concerns	🟢 Low	🟠 Some concerns	🔴 Serious risk

**Note.** Risk of bias in cohort studies was assessed using the ROBINS-I tool. Traffic light coding: 🟠 Some concerns = potential bias, but not critical; 🟢 Low = risk comparable to a well-performed randomized trial; 🟤Moderate = potential bias, unlikely to overturn conclusions; 🔴 High/Serious = substantial risk of bias, conclusions must be interpreted with caution.

**Table 6:** AHEI-2010 Dietary Guidelines and Relevance to Cancer Survivorship.

AHEI-2010 Component	Recommended Intake	Notable Findings
Vegetables	≥5 servings/day	Promotes survival
Fruits	≥4 servings/day	Supports antioxidant intake
Nuts and Soy Protein	≥1 serving/day	Linked to lower cancer mortality
White Meat/Fish to Red Meat Ratio	40 g:10 g per day	Improves fat quality in diet
Whole-Grain Fiber	≥15 g/day	Enhances metabolic control
Trans Fats	<0.5% of total energy	Reduces cardiovascular risk
PUFA/SFA Ratio	>1	Supports anti-inflammatory profile
Multivitamin Use	>5 years	Long-term usage may enhance resilience
Alcohol Consumption	1.5–2.5 drinks/day (men); 0.5–1.5 drinks/day (women)	Moderate intake aligns with MD

## Data Availability

No new data were generated or analyzed in this study. All data supporting the findings of this review are available within the published articles included in the systematic review. A full list of references is provided in the manuscript.

## Supplementary Materials

The following supporting information can be downloaded at: https://www.mdpi.com/article/10.3390/nu18050807/s1.

## Abbreviations

FRD	Fatigue reduction diet
HR	Hazard ratio
MD	Mediterranean Diet
RR	Risk ratio
OR	Odds ratio
OS	Overall survival
CR	Cancer Recurrence
CRF	Cancer-Related Fatigue
DFS	Disease-free survival
AHEI	Alternate Healthy Eating Index
PUFA	Polyunsaturated Fatty Acids
SFA	Saturated Fatty Acids

## Appendix A. Full Search Strategies

The complete search strings used for each database are provided below to ensure transparency and reproducibility.

PubMed

(“Mediterranean Diet” [Mesh] OR “Mediterranean dietary pattern” OR “MD adherence”)

AND (“Colorectal Neoplasms” [Mesh] OR “Colorectal Cancer” OR “CRC survivors”)
AND (“Recurrence” OR “Cancer-related fatigue” OR “Survival”)
Filters: Humans, Adults, English, 2000–2024

Embase

(‘mediterranean diet’/exp OR ‘mediterranean diet’ OR ‘dietary adherence’)

AND (‘colorectal cancer’/exp OR ‘colorectal tumor’)
AND (‘recurrence’/exp OR ‘fatigue’/exp OR ‘survival’)
AND [humans]/lim AND [adult]/lim AND [english]/lim

Scopus

TITLE-ABS-KEY (“Mediterranean diet” OR “Mediterranean dietary pattern”)

AND TITLE-ABS-KEY (“colorectal cancer” OR “CRC survivors”)
AND TITLE-ABS-KEY (“recurrence” OR “fatigue” OR “survival”)
AND (LIMIT-TO (LANGUAGE, “English”)) AND (LIMIT-TO (PUBYEAR > 1999))

Cochrane Library

(“Mediterranean diet” OR “Mediterranean dietary pattern”)

AND (“colorectal cancer” OR “CRC survivors”)
AND (“recurrence” OR “fatigue” OR “survival”)

## Appendix B. GRADE Summary of Findings Table

This appendix presents a GRADE-based summary of findings for the primary outcomes assessed in this systematic review: cancer recurrence and cancer-related fatigue (CRF). The GRADE approach evaluates evidence across five domains: risk of bias, inconsistency, indirectness, imprecision, and publication bias. Certainty of evidence was rated according to GRADE guidance, beginning at low certainty for observational studies and high certainty for randomized trials, with downgrading applied across domains as appropriate.

**Table A1:** GRADE Summary of Findings Table.

Outcome	No. of Studies	Study Design	Effect Estimate	Risk of Bias	Inconsistency	Indirectness	Imprecision	Publication Bias	Overall Certainty
Cancer recurrence	6	Prospective Cohort	HR = 0.83 (0.70–0.99)	Serious	Not serious	Serious	Serious	Undetected	⬤⬤⚪⚪ Low
Cancer-related fatigue	2	RCTs	MD = 0.29 (−0.58 to 1.16)	Moderate	Serious	Not serious	Very serious	Undetected	⬤⚪⚪⚪ Very Low
